# Supplementary material for: Road lighting density and brightness linked with increased cycling rates after-dark
Source: PLoS One. 2020 May 15;15(5):e0233105. doi: 10.1371/journal.pone.0233105 (PMC7228109; doi:10.1371/journal.pone.0233105)
Supplement: Attachment — Submitted filename: Response_to_Reviewers.docx [file pone.0233105.s008.docx]

**“Road lighting density and brightness linked with increased cycling rates after-dark” – Response to journal and reviewer comments**

| **Journal requirements** | | |
| --- | --- | --- |
| **ID** | **Comment** | **Response** |
| J1 | Please ensure that your manuscript meets PLOS ONE's style requirements, including those for file naming. | Supplementary file names have been edited to conform with requirements, and text updated in manuscript to reflect these changes. |
| J2 | We note that [Figures 1 and 3] in your submission contain [map/satellite] images which may be copyrighted. All PLOS content is published under the Creative Commons Attribution License (CC BY 4.0), which means that the manuscript, images, and Supporting Information files will be freely available online, and any third party is permitted to access, download, copy, distribute, and use these materials in any way, even commercially, with proper attribution. For these reasons, we cannot publish previously copyrighted maps or satellite images created using proprietary data, such as Google software (Google Maps, Street View, and Earth). For more information, see our copyright guidelines: http://journals.plos.org/plosone/s/licenses-and-copyright.  We require you to either (1) present written permission from the copyright holder to publish these figures specifically under the CC BY 4.0 license, or (2) remove the figures from your submission: | The map imagery used in Figure 1 has been changed to the OpenStreetMap Humanitarian layer. This layer has a CC0 license attached to it, which has no rights reserved and places the material fully in the public domain. No copyright agreement is required from the creator of the material. License details for the Humanitarian map layer of OpenStreetMap is available here: <https://github.com/hotosm/HDM-CartoCSS/blob/master/LICENCE.txt>  Text has been added to the Figure caption to reflect this CC0 license status of the map imagery used.  CC BY 4.0 licensing for Figures 3a and 3b is difficult to obtain. These images were informative but not crucial for the content of the paper, and have been removed. The numbering of subsequent Figures, including their captions and file labels, have been updated to reflect the removal of Figures 3a and 3b. |
| **Reviewer 1 comments** | | |
| **ID** | **Comment** | **Response** |
| 1.1 | The aim/objectives need to be stated clearly at the end of the introduction. Hypotheses ought to be listed at the end of introduction and not in the methods. | Aim of paper and hypotheses have been moved to end of Introduction (lines 142-151). |
| 1.2 | Similarly, the statements about the use of more precise quantitative definitions of lighting (lantern density and relative brightness) at the end of the introduction need to be moved to the methods. | These statements have been moved to become the opening paragraph of the Analytical strategy subsection in the Method section (lines 155-160). |
| 1.3 | In the methods, justification for choosing (22:00-22:59) as opposed for example to (21:00-21:59), which is also I assume dark throughout the year, is needed. | Text added to justify selection of the control hours (lines 198-203) – they were the latest / earliest hours that were consistently in daylight or darkness throughout the whole year. For example, 21:00-21:59 would partly occur before sunset for a portion of the year, at the location where the cycle count data were collected. |
| 1.4 | A segmentation of the analysis by areas/roads that are popular with cyclists vs those that are less popular would be useful to partly address at least one of the limitations discussed by the authors. | Further analysis added in Discussion to look at correlation between popularity of a counter location (as measured by total counts during the daylight control hour, 14-15:00) and odds ratio calculated at that location, for twilight and dark conditions (lines 657-663). New Figure added (Fig 7) to show these correlations. This analysis confirms no correlation between overall popularity of a location (i.e. counts during daylight) and odds ratio.  Please also note that in calculating these correlations using total counts in the daylight control hour, we found a slight error in the data used to calculate correlations with lantern density and pixel brightness – counts during the daylight control hour when the case hour was in daylight had been used, rather than across the whole year. The correlation statistics have been updated using total counts across the whole year (lines 654-657). This correction does not change the conclusion that there is no correlation between lighting and the popularity of a location. |
| **Reviewer 2 comments** | | |
| **ID** | **Comment** | **Response** |
| 2.1 | I wasn't sure why only one case hour was chosen. Why wasn't a morning case hour used as well? If this was done, and similar results were found for the two case hours, that would be supportive evidence. | We chose to only use an evening case hour because the transition between light conditions in an evening case hour over the course of a year is continuous – beginning in January it transitions from darkness to twilight, to daylight, to twilight, and back to darkness up until December. The transition in light conditions is not continuous in a morning case hour however, due to the biannual clock changes, which adds a layer of complexity and possible interpretation to results. For example, assume 06:00-07:00 was chosen as a morning case hour. The transitions between light conditions in this hour would look like this, for the year of 2015, in Birmingham, UK:  01/01/2015 – 24/02/2015 – DARK  25/02/2015 – 11/03/2015 – TWILIGHT  12/03/2015 – 28/03/2015 – DAY  29/03/2015 – 06/04/2015 – TWILIGHT  07/04/2015 – 08/09/2015 – DAY  09/09/2015 – 29/09/2015 – TWILIGHT  30/09/2015 – 24/10/2015 – DARK  25/10/2015 – 03/11/2015 – TWILIGHT  04/11/2015 – 31/12/2015 - DARK  We have added text to the Method to explain this and why we only used an evening case hour (lines 205-216), and we have added text to the Discussion to highlight that future work could look at using a morning case hour, and whether it does show the same results as found in the current analysis (lines 622-628). We have also added an additional reference (Sullivan & Flannagan, 2007), to highlight that other researchers using similar methods to examine effects of changes in ambient light have also only examined transitions in the evening, for similar reasons to those outlined above. |
| 2.2 | It would be interesting for the authors to discuss (or note) the issue of whether the low level of lighting needed to encourage riding in the dark would be sufficient to allow safe cycling. It might not be a good idea to encourage riding that was objectively high risk. | The reviewer is correct that lighting also plays a role in keeping cyclists safe, and we have added text to acknowledge this (lines 768-775), along with additional references. |
| 2.3 | A second minor issue is that the authors make the point that cycling is good for climate because it saves energy. Is there a trade-off, then, if more energy needs to be used for lighting in order to increase cycling? | The reviewer makes a good point. We have added text to the Conclusion that highlights the need to balance the potential benefits of lighting in terms of encouraging more cycling. We have also added estimates of the potential carbon costs of additional lighting in comparison to the carbon costs of driving, to highlight the balance towards lighting is favourabe. (lines 755-766). |
